# Lesion identification using unified segmentation-normalisation models and fuzzy clustering

**DOI:** 10.1016/j.neuroimage.2008.03.028

**Published:** 2008-07-15

**Authors:** Mohamed L. Seghier, Anil Ramlackhansingh, Jenny Crinion, Alexander P. Leff, Cathy J. Price

**Affiliations:** Wellcome Trust Centre for Neuroimaging, Institute of Neurology, UCL, London UK

**Keywords:** Structural MRI, Segmentation, Lesion identification, Gray matter, White matter, Oedema, Fuzzy clustering, Outlier detection

## Abstract

In this paper, we propose a new automated procedure for lesion identification from single images based on the detection of outlier voxels. We demonstrate the utility of this procedure using artificial and real lesions. The scheme rests on two innovations: First, we augment the generative model used for combined segmentation and normalization of images, with an empirical prior for an atypical tissue class, which can be optimised iteratively. Second, we adopt a fuzzy clustering procedure to identify outlier voxels in normalised gray and white matter segments. These two advances suppress misclassification of voxels and restrict lesion identification to gray/white matter lesions respectively. Our analyses show a high sensitivity for detecting and delineating brain lesions with different sizes, locations, and textures. Our approach has important implications for the generation of lesion overlap maps of a given population and the assessment of lesion-deficit mappings. From a clinical perspective, our method should help to compute the total volume of lesion or to trace precisely lesion boundaries that might be pertinent for surgical or diagnostic purposes.

## Introduction

Precise lesion identification in subjects with structural brain damage is essential for understanding lesion-deficit mappings in the human brain. Usually, the gold-standard method for lesion identification rests on the manual definition of abnormal brain tissue by a trained professional (e.g. Bates et al., 2003; Dronkers et al., 2004; Fiez et al., 2000); however, this method is laborious, operator-dependent (e.g. Ashton et al., 2003; Filippi et al., 1995), and time-consuming. Alternatively, several semi-automated and fully automated procedures for lesion identification have been proposed (e.g. Anbeek et al., 2004; Capelle et al., 2004; Colliot et al., 2006a,b; Datta et al., 2006; Fletcher-Heath et al., 2001; Hojjatoleslami and Kruggel, 2001; Mehta et al., 2003; Prastawa et al., 2004, 2003; Ruan et al., 2007; Sajja et al., 2006; Soltanian-Zadeh et al., 1998; Stamatakis and Tyler, 2005; Wu et al., 2006; Xie et al., 2005; Zhou et al., 2005). They can be divided in two categories according to the nature of the MRI images used to identify the lesion: (i) multi-channel or multi-spectral methods that operate on several weighted MRI images, including T1, T2, PD, and FLAIR images, with or without contrast agents (e.g. Kabir et al., 2007; Prastawa et al., 2004; Ruan et al., 2007; Soltanian-Zadeh et al., 1998; Wu et al., 2006); and (ii) mono-channel or mono-spectral methods that use only one contrast MRI image (e.g. T1 image) (Hojjatoleslami and Kruggel, 2001; Jack et al., 2001; Lau and Ozawa, 2006; Stamatakis and Tyler, 2005; Wilke et al., 2003).

With the financial and time constraints on clinical studies, it is not unusual that patients participating in functional MRI studies have only one anatomical image (usually a T1 image). Several algorithms have been proposed for extracting the maximum information from only one type of MRI contrast. Although these algorithms differ in terms of sensitivity, computational complexity and applicability, we can classify them according to two characteristics: (i) whether they use *a priori* knowledge about the structure of normal brains; including; for instance; a set of neurotypical templates or not and, (ii) whether they use tissue segmentation techniques or not. For example, voxel-based morphometry (VBM), which uses both template priors and segmentation, can characterise structural abnormalities in either the gray or white matter by segmenting and normalising MRI images of patients and comparing them to gray or white matter segments from controls (e.g. Colliot et al., 2006a; Gitelman et al., 2001; Kassubek et al., 2002; Wilke et al., 2003; Woermann et al., 1999). However, this approach has been shown to have low statistical sensitivity for detecting lesions (Mehta et al., 2003) and critically depends on the choice of the templates (Shen et al., 2007) and processing parameters (e.g. Wilke et al., 2003). Other groups have implemented alternative methods that eschew prior knowledge and segmentation. For example, the level-set evolution framework, with deformable models is based on cortical thickness and signal gradients (Colliot et al., 2006b; Ho et al., 2002; Xie et al., 2005). However, these approaches were developed to target specific lesion types (e.g., focal cortical dysplasia) and cannot be generalised easily to other types of lesions.

Recently, Stamatakis and Tyler (2005) proposed a lesion identification technique based on the comparison of the damaged brain to a set of normal brains without segmentation. The T1 images of controls and patients were normalised to the same template, spatially smoothed, and then compared statistically voxel-by-voxel to identify regions (i.e. lesions) that differed between patients and the normal range established by the controls (Stamatakis and Tyler, 2005). However, this approach depends on potentially suboptimal normalisation and the lesions identified can be contaminated by cerebrospinal fluid (CSF).

Another factor that may influence the success of automated lesion delineation is the location of the lesion. For instance, lesions close to the ventricles, the inter-hemispheric fissure, or to the scalp might be problematic, when using some automated methods (e.g., Liu et al., 2001; Stamatakis and Tyler, 2005). In this context, segmentation procedures might help to discriminate CSF and non-brain tissue before identifying the lesion. Furthermore, the existence of *a priori* knowledge about the distribution of normal values (assessed from normal controls) in all brain regions is very useful, particularly when the lesion has similar signal properties to healthy tissue (e.g. gray matter malformations (Bernasconi, 2003; Wilke et al., 2003)). These considerations suggest that robust frameworks for lesion detection should include tissue segmentation and *a priori* knowledge.

In this context, we propose a new procedure to identify any type of brain damage given a single anatomical image. Our procedure is based on the assumption that the lesion comprises atypical voxels that disclose themselves as outliers in gray and white matter segments. *Atypical voxels* are those that do not correspond to the expected tissue types; i.e., are neither grey matter (GM), white matter (WM), nor cerebrospinal fluid (CSF). To avoid misclassification, the segmentation routine has to be modified to account for the atypical “extra” tissue class introduced by the lesion. Here we propose a modified version of the unified segmentation scheme (Ashburner and Friston, 2005) to segment healthy and damaged brain tissue. The unified segmentation scheme has recently been shown to provide accurate spatial normalisation for lesioned brains (Crinion et al., 2007). *Outlier voxels* are those that are far from the normal range of voxel values in controls (Van Leemput et al., 2001). The lesion is then identified by searching for outlier voxels in GM and WM segments. This ensures that we identify lesions that are specific to brain tissue (i.e., GM and WM). We use fuzzy clustering with fixed-prototypes (FCP), developed recently for outlier detection in second-level functional MRI analyses (Seghier et al., 2007). A few studies have tried to implement this scheme (i.e., identify unexpected and outlier voxels) in multi-spectral mode (e.g. Prastawa et al., 2003, 2004), but its applicability has not yet been demonstrated in mono-spectral mode. In this paper, we evaluate the approach with structural T1 images.

The approach is illustrated using both simulated and real patient data. We first explain the rational for using a modified segmentation procedure. We show that adding an extra class to the unified segmentation model effectively precludes the misclassification of damaged voxels as healthy GM or WM tissue. Then, the success of the modified segmentation is assessed qualitatively by ensuring that (i) GM and WM are free from contamination from the lesion and (ii) the extra class does not incorporate intact tissue. Outlier GM and WM voxels are identified by fuzzy clustering and lesion maps are then generated. Using similarity measures, we explore the sensitivity and the specificity of our method for detecting and delineating brain lesions. The results from both our artificial and real lesions demonstrate successful delineation of damaged tissue with high sensitivity.

## Methods

### Data sets

#### T1 images with simulated lesions

Ten T1-weighted images from neurologically normal subjects were modified by inserting a variety of lesions that had been derived from “real” lesioned brains. Full details of how these T1 images were created can be found in Brett et al. (2001). Essentially, the simulations involved creating a lesion definition image (i.e., a manual definition of lesioned tissue) using T1-weighted MRI images of real patients' brains with a variety of lesions. These lesion images were then inserted, “cut-and-pasted”, into T1-weighted MRI images from normal subjects. The lesions were heterogeneous, of different size, T1 signal and location (see Brett et al., 2001). Simulated case 01 had metastasis with extensive oedema; 02 had a left anterior frontal infarct near to the scalp; 03 had left anterior and mesial communicating artery aneurysm and temporoparietal infarct; 04 had dorsal and mesial cortical dysplasia; 05 had a posterior and mesial left occipitotemporal infarct; 06 had a large left frontoparietal loculated infarct; 07 had left temporoparietal infarct; 08 had focal cortical atrophy affecting the temporal lobe; 09 had a posterior and mesial left occipitotemporal infarct and 10 had an infarct in the putamen/insula (for illustration, see Fig. 2 of Crinion et al. (2007)).

In contrast to some previous studies (e.g. Mehta et al., 2003; Stamatakis and Tyler 2005), we used simulated lesions that were derived from real cases rather than using artificial lesions that do not reflect the complexity of real lesions. There are two key advantages of simulated lesions: the natural characteristics, in terms of the size and texture of the lesion, are preserved and we know *a priori* where the boundaries of the lesion are.

#### Real T1 images

T1 images were acquired from eight patients with strokes that varied in size and affected diverse regions (age 23–68 years) and sixty-four neurologically normal subjects (age 21–75 years). To test the sensitivity of our method on a wide range of lesion volumes and locations, we included: two patients with large and heterogonous frontotemporal lesions extending laterally from the ventricles to near the scalp; two patients with large occipitotemporal lesions; two patients with left hemisphere lesions near to the ventricles; one patient with a right hemispheric lesion near to the ventricles; and one patient with a small infarct close to the inter-hemispheric fissure. These real cases illustrate some of the characteristics that might challenge automated identification of lesions.

All acquisitions were performed on a 1.5 T Siemens system (Siemens Medical Systems, Erlangen, Germany). Anatomical imaging consisted of a weighted T1 GRE 3D sequence (TR/TE/Flip = 12.24 ms/ 3.56 ms/ 23°, matrix = 256 × 256, in-plane resolution = 1 × 1 mm, 176 axial slices, 1 mm thick with no gap).

### Lesion identification procedure

Our approach comprises four steps:

• Segmentation and normalisation of all patients and controls T1 images (allowing for a lesion tissue class).
• Spatial smoothing of normalised GM and WM segments.
• Detection of outlier voxels in each tissue by comparing the GM and WM segments of the patient to those of controls under fuzzy clustering.
• Outlier voxels in each tissue class are assigned to the lesion (i.e., a fuzzy set).

Note that for illustration purposes we will use the simulated cases 10 and 06 to disclose the rational of each step of our automated method. Simulated case 10 has a chronic stroke affecting the insula (tissue loss) with well-defined borders and a relatively homogeneous T1 signal distribution (close to CSF signal). In contrast, simulated case 06 has a large frontoparietal, loculated lesion with diffuse borders, local mass effects and a heterogeneous T1 signal distribution (tissue loss with T1 signal near to CSF and tissue damage with T1 signal near to GM).

#### Segmentation of the T1 volumes

##### Unified segmentation-normalisation

**Fig. 1:**
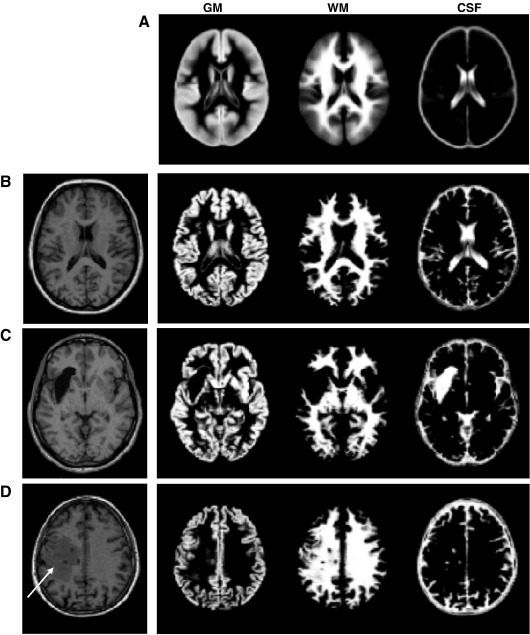
(A) GM, WM, and CSF priors as predefined in SPM5. Segmentation into GM, WM and CSF tissues of normal T1 volume (B), simulated case 10 (C), simulated case 06 (D) with the standard unified segmentation routine. Chronic lesion in simulated case (10) is segmented in the CSF class. The presence of damage in simulated case 06, misclassified as intact WM (D), is indicated with a white arrow.

We employed the unified procedure implemented in the SPM5 software package (Wellcome Trust Centre for Neuroimaging, London, UK, http://www.fil.ion.ucl.ac.uk/spm/) that combines segmentation, bias correction and spatial normalization through the inversion of a single unified model (for more details see Ashburner and Friston, 2005). In brief, the unified model combines tissue class, intensity bias and nonlinear warping into the same probabilistic models that are assumed to generate subject-specific images. Image intensities are modelled by mixtures of Gaussians (MOG). Within a MOG, the prior probability that a voxel intensity is drawn from a particular Gaussian is given by a mixing proportion. In the unified model, the priors on the tissue class from which intensities are drawn are encoded by deformable tissue probability maps. These are generated from the averages of affine registered and tissue classified images of 452 subjects (see Fig. 1A), provided by the International Consortium for Brain Mapping (http://www.loni.ucla.edu/ICBM/). These maps represent the probabilities of finding GM, WM, CSF and “other” tissues at each voxel. In this paper, the unified model used the default number of Gaussians {2,2,2,4} to model each of the intensity distributions of GM, WM, CSF and the “other” tissues respectively.

Throughout this work, the following parameters of the SPM5 scheme were held constant: 25 mm for the cut-off of three dimensional discrete cosine transform (DCT) basis functions for spatial warping (for more details see Ashburner and Friston, 1999), medium regularisation (see Crinion et al., 2007), and 75 mm width for the Gaussian smoothness of intensity bias fields.

##### Problems with the segmentation procedure

To illustrate the effectiveness of the unified segmentation using the three (plus “other”) priors shown in Fig. 1A, a normal brain and two simulated lesions (cases 10 and 06) were processed using the SPM5 segmentation routine. The normal brain was segmented as expected into GM, WM and CSF (Fig. 1B). In simulated case 10, the well-defined insular lesion is entirely classified as CSF (Fig. 1C), despite the fact that the normal priors were higher for WM than CSF at this spatial location. The lesion site was therefore classified with a low probability of being GM or WM which is important when identifying areas with abnormally low GM or WM. In contrast, for simulated case 06, a part of the lesion was misclassified as normal WM tissue (Fig. 1D), despite the fact that this tissue had a T1 signal different from the T1 signal in healthy WM. This cannot entirely be explained by the mixing of two Gaussians because the same area remained misclassified as WM even when using one Gaussian per class to model the signal distribution of each tissue. This suggests that the relatively high WM priors at this spatial localisation had biased the apparent difference in T1 signal during the optimisation procedure (for more details, see Eq. (5) in Ashburner and Friston, 2005). For our purposes, this segmentation failure will hinder lesion identification. This problem has been documented previously in patients with focal cortical dysplasia, where parts of the lesions were misclassified as intact WM (see for example Colliot et al., 2006a). A modification of the segmentation procedure therefore needs to ensure that abnormal voxels are not misclassified as GM or WM.

##### A modified segmentation procedure

One simple solution to the problem of misclassification of damaged tissue entails adding an “extra” class that includes the unexpected and abnormal voxels within the lesion. This is equivalent to the segmentation procedure in some multi-spectral studies that use the regions that show signal enhancement after contrast agent injection as priors (e.g. Moon et al., 2002; Prastawa et al., 2003). Although lesion location, shape, size, and signal are unknown, we show below how the priors can be approximated for the extra class in a mono-spectral mode. Before describing this extra class, we illustrate its rational by displaying the segmented images in a new way. This involves generating signal-to-probability maps that represent a scatter-plot (i.e., a voxel-by-voxel correspondence) between the T1 image (signal) and the probability of a segmented tissue type.

**Fig. 2:**
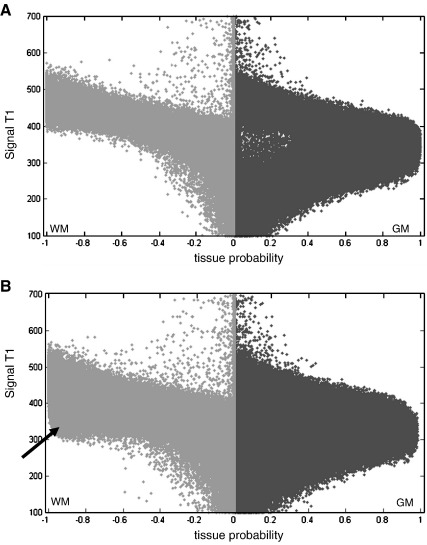
Signal-to-probability maps of a normal brain (A) and simulated case 06 (B). These maps represent a scatter-plot between the T1 image and the segmented tissue. T1 values are coded in an arbitrary unit (from 100 to 700). The GM probability is shown on the right of each plot (dark gray) and the WM probability is shown on the left (light gray). For display purposes, the WM probability values are multiplied by − 1. The black arrow indicates the misclassified tissue in the WM.

Fig. 2 illustrates a scatter-plot of a normal brain. Higher probability values (around 1) for WM and GM corresponded to different T1 signal ranges, suggesting a good partition between the two tissues. However, in the presence of a lesion with a heterogeneous signal, as in simulated case 06, high WM probability was also observed for voxels with a T1 signal range similar to those of the GM class (voxels indicated by an arrow in Fig. 2B). This suggests that the WM class actually contained voxels from the lesion with a T1 signal far from the normal WM signal; i.e., the WM class has become over inclusive.

The modified segmentation algorithm aims to suppress over inclusive WM and GM classes, especially when the lesion has a T1 signal in the range of WM or GM signal. By adding an extra class to the segmentation routine, we aimed to model explicitly voxels that show a discrepancy between the spatial priors and the expected T1 signal (e.g. a location in the WM but with T1 signal values close to GM). To suppress misclassified voxels (those indicated by an arrow in Fig. 2B), the priors for this extra class should exhibit the following characteristics. First, as the T1 signal in the lesion (e.g. oedema, dysplasia) and GM are comparable, the extra class should have a low probability in GM voxels to avoid incorporating normal GM voxels. Similarly, to differentiate misclassified and intact WM voxels, the extra class should have a low probability in WM voxels to ensure that normal WM are classified properly; however, as misclassified voxels are particularly evident within WM, the probability of the extra class should be higher for voxels located in WM, relative to GM, to “force” the segmentation to reclassify the misclassified tissue. Although these criteria can be satisfied by many priors for the extra class, one simple prior is the mean of WM and CSF priors:

(1) $$P_{extra}=\frac{P_{WM}+P_{CSF}}{2}\text{.}$$

Where *P*_*WM*_ and *P*_*CSF*_ are the standard priors of WM and CSF respectively (as shown in Fig. 1A). In short, a new class is formed by taking prior probability mass from the WM and CSF classes but not the GM class (note that *P*_*extra*_ values in GM tissue are not zeros). This extra class provides more flexibility in the segmentation procedure, when dealing with the damaged tissue as illustrated below.

##### Implementing the modified segmentation

**Fig. 3:**
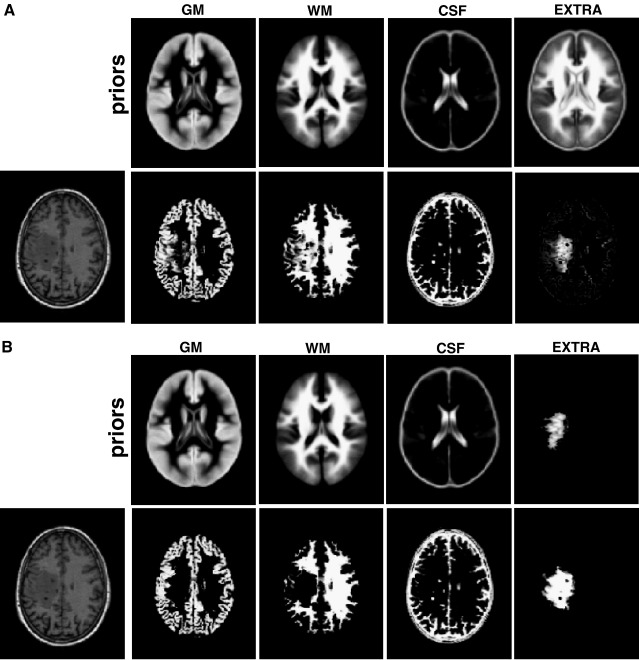
(A) modified segmentation of simulated case 06: (top) the 4 prior images; (bottom) the segmented tissues (GM, WM, CSF) and a first approximation of the extra class. (B) modified segmentation of the same case using the result of the first segmentation run as priors on the extra class. Note that the damage (i.e. spatially located in WM but with T1 signal close to GM) is now classified in the lesion class and not as intact WM.

Fig. 3 illustrates how the modified segmentation routine dealt with damaged tissue in simulated case 06. By adding this extra class, the segmentation was able to classify the abnormal WM tissue (see Fig. 3A). Although most of the abnormal voxels were given a low probability of being intact WM (as can be seen when comparing Fig. 3A to Fig. 1D), some of the abnormal voxels remained misclassified as WM or GM. To improve the segmentation procedure further, the extra class was refined and then used as an empirical prior for a second segmentation on the same T1 image. To obtain a refined version of the extra class, all the voxels classified in the extra class with a probability value lower than a third were removed. This ensured that, at the same location, voxels belonging to the extra class would have a higher probability value than either GM or WM. When using this refined version of the extra prior, the second segmentation removed almost all abnormal voxels from the WM class (see Fig. 3B for more details). This is clearly visible when comparing the WM classes in Figs. 1D and 3B. Remarkably, this segmentation procedure, with two iterations and appropriate extra class priors, modelled abnormal voxels and thus minimised misclassification in GM and WM classes. Note that, at each iteration, the segmentation routine of SPM5 ensures that all priors sum to one at any voxel.

Practically, the process shown above can be iterated several times (i.e., an iterative segmentation routine): the estimated extra class acting as the prior for the next segmentation run. Here we use two iterations throughout the paper. For more details about the influence of the number of iterations, see paragraph 1 and Fig. S1 of the Supplementary material.

**Fig. 4:**
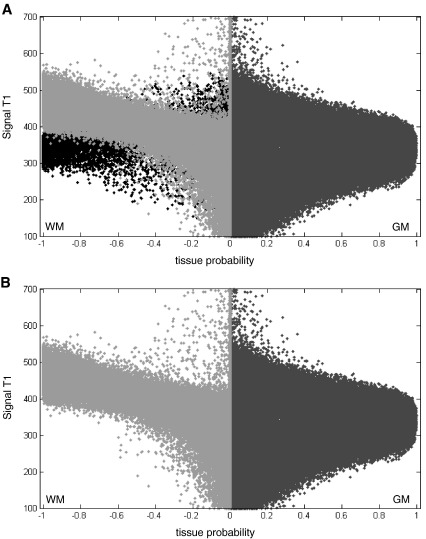
(A) Signal-to-probability maps of simulated case 06. These maps represent a scatter-plot between the T1 image and the segmented tissues. T1 values are coded in an arbitrary unit (from 100 to 700). The GM probability is shown in dark gray and the WM probability is shown in light gray. For display purposes, the WM probability values are multiplied by − 1. Voxels located in the WM but correctly classified as lesion (in the extra class) are shown in black. (B) After removing the voxels inside the lesion (those shown in black in A), the signal-to-probability maps of simulated case 06 are comparable to those of a normal brain (both GM and WM tissues are no longer contaminated by the lesion).

With two iterations, Fig. 4 shows clearly how adding an extra class improved the dissociation of normal and abnormal WM voxels. In these maps, the abnormal WM voxels (shown in black in Fig. 4A) are classified in the extra class. The final WM class is remarkably similar to a normal WM class (e.g. the scatter map of a normal subject in Fig. 2A was comparable to the one of the correctly segmented lesioned brain in Fig. 4B). Critically, we also found that adding this extra class did not alter the segmented healthy tissue when the brain is normal (for more details see paragraph 2 and Fig. S2 of the Supplementary material).

This new segmentation approach which creates 4 normalised and segmented classes per subject (i.e., normalised GM, WM, CSF and the extra class) was applied to ten brains with simulated lesions and eight brains with real lesions. For the purpose of lesion identification, only the GM and WM images were used (i.e., here we are only interested in lesions of gray and white matter).

#### Spatial smoothing

Before comparing the segmented images of a lesioned brain to those of controls, we need to suppress fine-scale inter-subject anatomical variability. For this purpose, the normalised and segmented GM and WM images of each subject were smoothed spatially with a Gaussian kernel of 8 mm full-width-at-half-maximum (FWHM). See paragraph 3 and Fig. S3 of the Supplementary material for more details about our reasons for selecting 8 mm smoothing. This is in line with other studies that have shown better results with intermediate smoothing values (8–12 mm) for lesion identification (e.g. Stamatakis and Tyler, 2005).

#### Fuzzy clustering of outliers

To identify outlier voxels, we have described previously a method based on Fuzzy Clustering with fixed Prototypes (FCP). This has been used to identify voxels where activation in a given subject is far from the mean activation of the group or population (for more details, see (Seghier et al., 2007). In the context of lesion identification, we assume that, at the global level, a lesioned brain can be considered as an outlier in relation to normal (control) brains. This assumption has been suggested for instance in some previous multi-spectral work (e.g. Jack et al., 2001; Prastawa et al., 2004; Van Leemput et al., 2001). Here, we used FCP to identify voxels that were very different in the lesioned brain as compared to controls.

The procedure is as follows: a lesioned GM segment is compared with control GM segments for each voxel *i*, by assessing a similarity metric *D*_*ij*_ that represents the difference between the value of voxel *i* of a *j*-th GM fuzzy set and the mean of GM values in this voxel (with *j* = 1… *N*_*sub*_; *N*_*sub*_ is the total number of subjects (i.e. number of controls plus the patient)). The similarity metric *D*_*ij*_ is defined as:

(2) $$D_{ij}=1-\tanh(\frac{N_{sub}}{N_{sub}-1}\cdot \frac{X_{ij}-{\overset{―}{X}}_{i}}{\alpha })\text{.}$$

The real constant *α* is a “tuning” parameter that can be adjusted to control the sensitivity of the method to outlier values (see Fig. 3 of Seghier et al. (2007)). *tanh* is the hyperbolic tangent, *X*_*ij*_ is the tissue probability for the *j*-th subject at voxel *i*, and *X̄*―_*i*_ is the mean over subjects. The constant α is fixed at − 0.5 (the constant *α* is negative to test if *X*_*ij*_ is low compared to *X*―_*i*_, which is in line with the definition of a lesion as an absence of normal brain tissue).

The similarity metric *D*_*ij*_ is then used to quantify the degree of membership *U*_*ij*_ of voxel *i* to class *j* according to the following equation:

(3) $$U_{ij}=\frac{D_{ij}^{\lambda }}{\sum_{j}D_{ij}^{\lambda }}\text{.}$$

The parameter *λ* is a negative number, typically − 4. The values *U*_*ij*_ (within the interval [0,1]) comprise the *j*-th fuzzy set. When *j* indexes the lesioned brain, the values *U*_*ij*_ comprise the fuzzy set *F*_*GM*_, which represents the degree of membership of voxels that have very low GM probability in the lesioned brain, relative to controls (i.e., a GM lesion map). This procedure was repeated with WM images, yielding the fuzzy set *F*_*WM*_.

#### Grouping GM and WM lesions

From the above, we obtain two fuzzy sets *F*_*GM*_ and *F*_*WM*_, representing the voxels in each lesioned brain that had a very low probability of being GM and WM voxels respectively, compared with the controls. The union, *F*_*LES*_ of the two sets identifies the lesion (i.e., a probability of being either GM or WM). The fuzzy set union was assessed with (c.f., Ibrahim, 1997),:

(4) $$F_{LES}=F_{GM}\cup F_{WM}=\max(F_{GM},F_{WM})$$

where ∪ is the set operator “union”. The operator “max” is applied over all voxels.

**Fig. 5:**
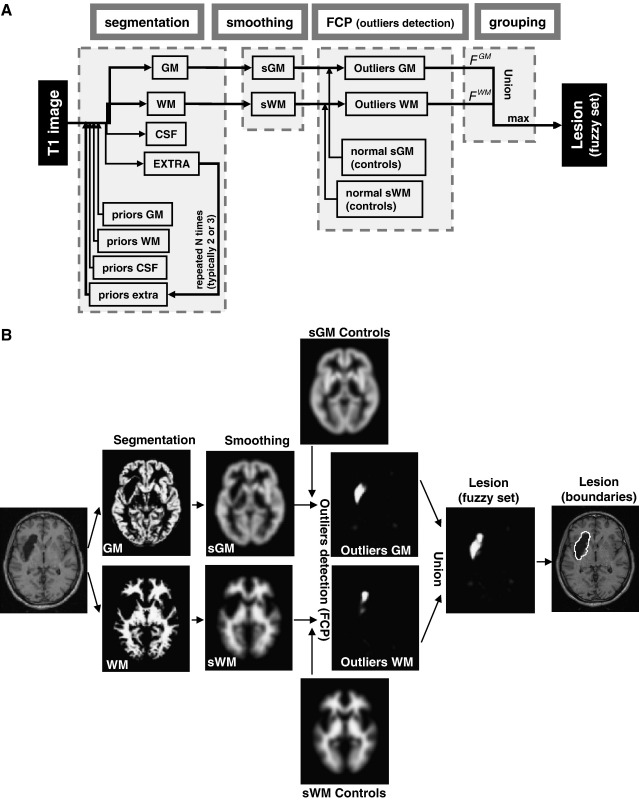
(A) a schematic view of the different steps, from segmentation to grouping, of our automated lesion identification method. (B) An illustration of the resulting images for simulated case 10 using the modified procedure.

A schematic view of our scheme is provided in Fig. 5A. An illustration of this procedure is given in Fig. 5B with simulated case 10. Briefly, after segmentation, the tissue images were smoothed (with FWHM of 8 mm) and compared to normal tissue images from the control subjects to identify outlier voxels. These GM and WM outlier voxels are then combined to form the fuzzy set that defines the lesion. This fuzzy set can be thresholded at a given *U* value to generate a binary mask of the lesion (for more details about the influence of *U* thresholds, see paragraph 4 and Fig. S4 of the Supplementary material).

### Validation of the identified lesions

At the global level, the method is judged successful if it can identify the lesion at the right location and with approximately the correct extent. At the voxel level, we assessed Dice's similarity index (Dice, 1945) between each binary lesion map (i.e. *F*_*LES*_ at a given *U* threshold) and the “real” lesion (considered as true positives) using the following formula:

(5) $$Dice=\frac{2\cdot TP}{2\cdot TP+FP+FN}$$

where TP, FP, and FN represent the number of true positives, false positives, and false negatives respectively (for a similar rational see Sajja et al., 2006; Stamatakis and Tyler, 2005). Because the lesion is a fuzzy set (i.e. *F*_*LES*_ contained values between 0 and 1), the Dice index was generated at several *U* thresholds (c.f. Anbeek et al., 2004). To assess the specificity and the sensitivity of our method, we also generated receiver operating characteristic (ROC) curves (e.g. Metz, 1978) that encode the dependence of the true positive rate (sensitivity) on the false positive rate (one minus specificity) for different *U* thresholds.

For simulated lesions, the “real” (true) abnormal voxels are approximately known (inserted with the cut/paste procedure as explained above). For real cases, all lesions were manually segmented by an expert (A.P.L) and the resultant masks are thus considered as true voxels.

## Results

### Definition of simulated lesion boundaries

**Fig. 6:**
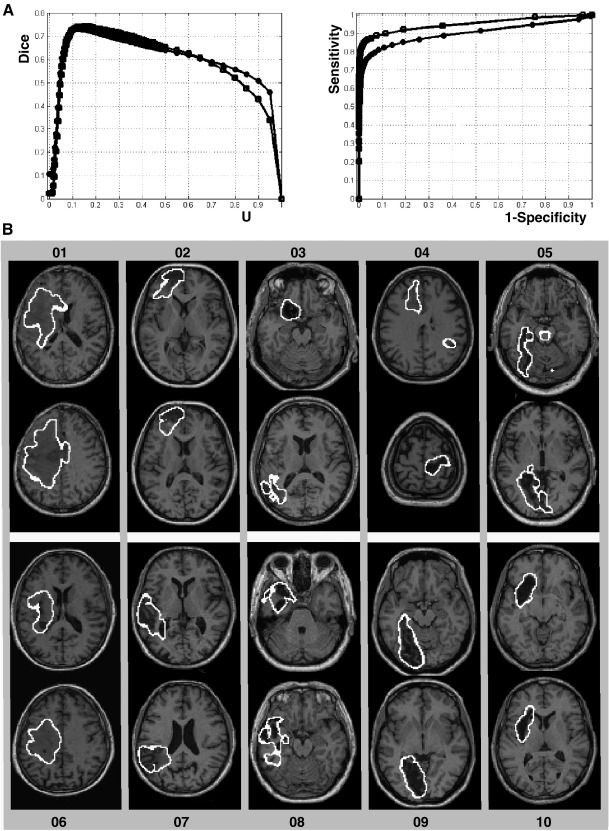
(A) For simulated cases 06 (circles) and 10 (squares), illustration of the Dice index (left) at different *U* thresholds and ROC curves (right) that plot the true positive rate (sensitivity) on the false positive rate (one minus specificity) for different *U* thresholds. (B) Axial slices illustrating the lesion boundaries of all simulated cases.

At the voxel level, we illustrate the sensitivity of the method on simulated cases 06 and 10 (those presented in Fig. 1C and D). Fig. 6A shows the Dice similarity index at different *U* thresholds. At low *U* values (e.g. *U* < 0.05), the Dice index was small due to high false positive rates. The Dice index reached high values (> 0.7) at intermediate *U* values suggesting a remarkable correspondence between the identified lesions and the known simulated lesions. The method is also highly sensitive and specific as illustrated by the ROC curves (i.e. curves near to the top-left corner). At the global level, the boundaries of all simulated lesions are shown in Fig. 6B. All lesions were identified successfully, including the extensive oedema in simulated case 01, both the aneurysm and infarct in simulated case 03, dysplasia in simulated case 04, large tissue loss and tissue damage in simulated case 06 and atrophy in simulated case 08. Critically, although a low *U* threshold was used (*U* = 0.1) in Fig. 6B, false positives (i.e., intact tissue identified as damaged) were very limited (e.g. brainstem of simulated case 05 and WM tracks in simulated case 07).

### Definition of real lesion boundaries

**Fig. 7:**
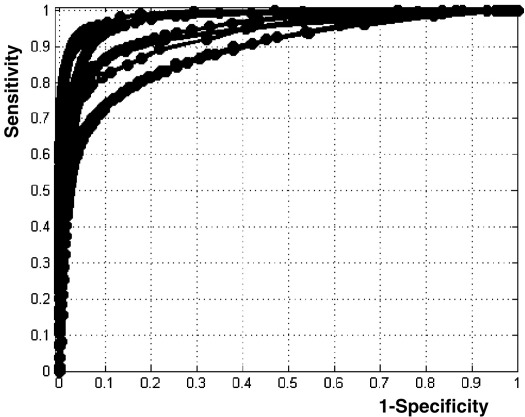
ROC curves of the eight real cases. All curves are remarkably close to the top-left corner (i.e. near to the manual segmentation).

**Fig. 8:**
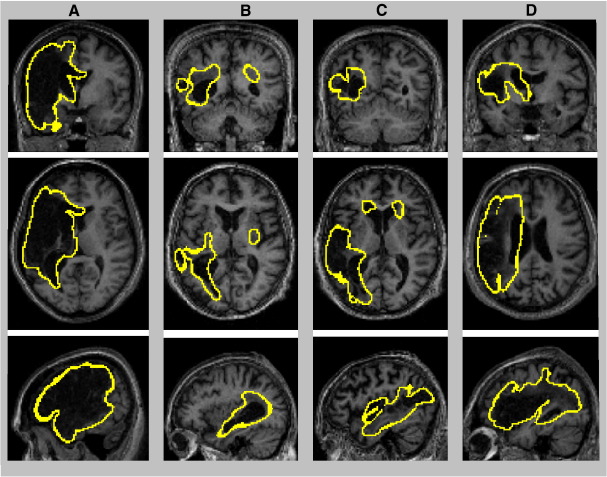
Illustration of the boundaries of four real cases with large and heterogeneous lesions on coronal, axial, and sagittal views. Lesion boundaries are displayed at a threshold of *U *> 0.3 (threshold applied on the fuzzy lesion set).

Given real patient scans, the method was able to identify automatically, and without exception, a wide range of lesions. Fig. 7 shows the ROC curves of the eight real cases. Compared to the manual segmentation (i.e. that defines the “true” abnormal voxels), our method is highly sensitive and specific for these different lesions. The Dice index is on average 0.64 ± 0.1 with a maximum at 0.81. One real case has a small Dice index (0.53) due to non-overlapping voxels between the manual segmentation and our method, in particular near to the ventricles (see Fig. 8C below), despite lesions identified at the right location.

**Fig. 9:**
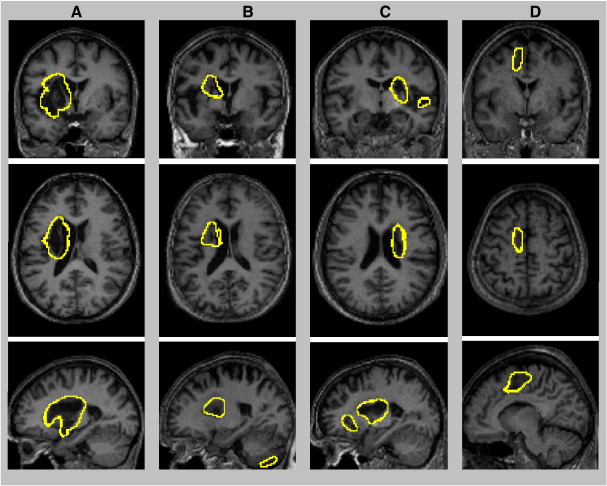
Illustration of the boundaries of three real cases with lesions near to the ventricles (A–C) and one real case with a lesion near to the inter-hemispheric fissure (D) on coronal, axial, and sagittal views. Lesion boundaries are displayed at a threshold of *U* > 0.3.

Patients with extensive lesions are shown in Fig. 8. In these four cases, lesions were identified correctly, including massive tissue loss (Fig. 8A) and extensive tissue damage in different parts of the brain (Fig. 8B–D). More challenging cases were the patients with tissue loss near to the ventricles (i.e., comparable T1 signal in lesions and ventricles). Fig. 9 shows the results in three patients; the boundaries of the lesions appear well distinguished from the ventricles, which confirms that our procedure minimised any contamination from ventricles during lesion identification. Lesion identification was successful in both left (Fig. 9A–B) and right hemispheres (Fig. 9C). The last real case is shown in Fig. 9D. The method was remarkably successful in this case, despite a lesion near the inter-hemispheric fissure (Fig. 9D).

## Discussion

In this paper, we propose a new automated procedure for lesion identification from single images based on the detection of outlier voxels. We have demonstrated the utility of this procedure on multiple artificial and real lesions. Our findings show the high sensitivity of the method for detecting and delineating brain lesions with different sizes, locations, and textures. Our approach has important applications for the generation of lesion overlap maps of a given population (e.g. Frank et al., 1997; Makale et al., 2002) and the assessment of lesion-deficit mappings (Bates et al., 2003; Damasio et al., 2004; Karnath et al., 2004; Solomon et al., 2007; Tyler et al., 2005).

The rational for using this method is motivated by the following points: (i) this method is suitable when only one type of image (e.g., T1 image) is available; (ii) intensities in the segmented classes are automatically normalised into the interval 0 to 1 for each subject, which should minimise the influence of MR signal non-uniformities (Hou, 2006) that may hinder the detection of lesion from T1 volumes (e.g. Stamatakis and Tyler 2005); (iii) the analysis was performed on voxels within the tissues of interest (GM and WM) avoiding contributions from the CSF and non-brain classes; (iv) it can assess the lesion effect specifically for each brain tissue; e.g., GM malformations; (v) the existence of the lesion is modelled explicitly as an extra class during brain segmentation, which helps to avoid misclassification of damaged voxels; (vi) identified lesions are mapped directly in a stereotaxic space (e.g. Rorden and Brett, 2000); (vii) the algorithm is based on optimal normalisation (i.e. the unified segmentation–normalisation framework) that has been shown to be accurate and robust when dealing with lesioned brains (Crinion et al., 2007); (viii) the algorithm for outlier detection is based on the pragmatic theory of fuzzy sets; (ix) the categorisation of a voxel as intact or abnormal is based on the distribution of normal values of GM and WM in healthy subjects; (x) identified lesions are coded as continuous values thereby quantifying the degree of abnormality of each voxel.

Some methodological factors may limit the sensitivity of our method. The spatial smoothing used here to minimise inter-subject anatomical variability during the identification of outliers obviously has an influence on the sensitivity and specificity of the method. In line with previous reports (e.g. Stamatakis and Tyler, 2005; Wilke et al., 2003), intermediate smoothing values (e.g. around 8 mm of FWHM) appear to be suitable for the size of lesions tested here. However, this parameter should be adapted to match the size of the expected lesions (due to the matched filter theory (Salmond et al., 2002a; White et al., 2001)), for instance by using low smoothing if the method is applied to identify tiny lesions (e.g. subcortical lesions (Vogels et al., 1995) or subtle GM malformations (Kassubek et al., 2002)). In addition, the sensitivity of the method also depends on the value of the parameter α during the quantification of the similarity measure *D* (i.e. see Eq. (2)). Basically, the parameter α controls how far a voxel should be from the mean of controls before it is considered an outlier. Here, it was set objectively to midway in the range of possible probability values (i.e. half the interval [0,1]). An extensive discussion of the influence of the parameter α can be found elsewhere (Seghier et al., 2007).

Critically, the comparison between the brain-damaged subjects and healthy subjects assume that both groups differ only according to the presence or absence of abnormal tissue. Thus, it is important to cancel or minimise all other potential sources of differences that might be caused by demographic variables, including age and gender. For instance, comparing an elderly subject to younger controls may lead to the detection of abnormal voxels caused by structural differences outside the real lesion (although atrophy detection might be relevant in other contexts; e.g. Convit et al., 2000). In addition, the specificity of the method also depends on the accuracy of the segmentation and the normalisation procedure. The normalisation procedure should accurately match the damaged brain to the controls to ensure good correspondence of healthy tissues in both groups. Several parameters may influence the quality of the normalisation procedure (e.g. Robbins et al., 2004; Salmond et al., 2002b; Shen et al., 2007; Wilke et al., 2003). Here we used an optimal normalisation procedure that has recently been shown to be more accurate in brain-damaged subjects than standard procedures (Crinion et al., 2007).

During the evaluation of our method, we assumed manual segmentation as the gold-standard method in order to define the “ground-truth” (true abnormal voxels) used for computing the Dice similarity index and evaluating ROC curves. However, this does not guarantee that misclassified voxels will not be included in the manually segmented lesions (which will lead to an increase of false positives/negative). For instance, Fiez et al. (2000) estimated the percentage of intra- and inter-operator errors during lesion identification in 10 subjects. In particular, they showed that, at the voxel level, the intra-operator error (i.e. percentage of nonoverlaping voxels between two lesion segmentations of the same subject and by the same operator) was on average 26% to 36% (for more details see Table 3 of Fiez et al. (2000)). Note also that the definition of “exact” lesion boundaries in a mono-spectral mode is difficult even with manual segmentation, because of partial volume effects in T1 images with limited spatial resolution (e.g. Guttmann et al., 1999; Links et al., 1998).

In this paper, we tested our method on a variety of lesions (size, location, texture). However, it is likely that other kinds of lesions might be challenging. Some lesion locations might be more difficult; for instance, posterior fossa lesions (e.g. infratentorial tumors) can be difficult to identify automatically (Gass et al., 2000). As the gyral anatomy of the cerebellum is more convoluted than that of the cerebrum, lesions in this region may give rise to disproportionate errors of spatial normalization. In this context, it might be useful to use more accurate cerebellar templates (for a similar rational see Diedrichsen, 2006). Other lesion locations or causes of abnormal signal intensity may also be problematic for our method. Periventricular lesions (lesions contiguous with ventricles, e.g. Zimmerman et al., 1986) can be classified as WM lesions but they are very difficult to distinguish from atrophy effects or CSF-partial voluming (Payne et al., 2002). In all these cases and as a general principle we recommend that all automated lesion profiles be checked by eye.

Finally, as shown above, identified lesions are coded as continuous values (the fuzzy degree of membership *U* in the interval [0,1]) providing a useful quantification of the degree of abnormality of each voxel. This is essential for methods that use continuous lesion values for generating lesion-deficit mappings (e.g. Tyler et al., 2005). On the other hand, for other mapping methods (e.g. Bates et al., 2003; Damasio et al., 2004; Dronkers et al., 2004; Karnath et al., 2004) or lesion overlap methods that use binary lesions, the binarisation process can be performed by applying a *U* threshold (typically 0.3) to the fuzzy lesions.

In summary, we have presented an automated method for lesion identification in mono-channel MRI that can be implemented easily within the SPM5 software package. With a modified segmentation procedure and fuzzy clustering, our approach was able to detect and delineate a variety of artificial and real lesions. From a clinical perspective, our method should help to compute the total volume of lesion, when this measure is appropriate, or to trace precisely the frontiers between the intact and damaged tissue that might be pertinent for surgical or diagnostic purposes.
