# Supplementary material for: Lesion identification using unified segmentation-normalisation models and fuzzy clustering
Source: Neuroimage. 2008 Jul 15;41(4-3):1253–66. doi: 10.1016/j.neuroimage.2008.03.028 (PMC2724121; doi:10.1016/j.neuroimage.2008.03.028)
Supplement: Supplementary file 1 [file mmc1.pdf]

1- Influence of the number of iterations

Figure S1A illustrates the evolution of the priors for the extra class when repeating the segmentation routine several times. One observation is the increase of abnormal tissue extent in the priors (as shown for simulated case 06). However, because it was not possible to define morphological constraints on the extent of the abnormal tissue at higher iterations, the priors of the abnormal tissue wrongly included some normal GM voxels (principally due to closer T1 signal between the abnormal voxels in the extra class and the normal voxels in the GM). Critically, the probability that healthy GM in the vicinity of the lesion is classified in the extra class (i.e., as damaged tissue) increased with the number of iterations (for more details see Figure S1C).

This also demonstrated the increased False Positive Rate (FPR; i.e. 1-specificity) as a function of the number of iterations (Figure S1B). The FPR is defined as:

$$FPR = \frac{FP}{FP + TN}$$

where FP and TN represent the number of false positives and true negatives respectively. To avoid this artefact, a small number of iterations (<5 iterations; e.g. FPR<2% for simulated case 06) appeared to be a reasonable compromise for a better definition of the extra class, without altering classification of normal tissue.

In principle, one could use the evidence of the unified model, at each iteration, as an objective function to optimise the empirical priors. This is possible because we are using an explicit generative model for the segmentation and it is possible (in principle) to integrate out dependencies on the model parameters to provide the evidence of likelihood of the data under each empirical prior. We will pursue this elsewhere and assume that two iterations is near-optimal for our purposes.

2- Segmentation of the healthy tissue

We also investigated whether adding this extra class altered the segmented healthy tissue when the brain is normal. On a subgroup of 8 normal brains, we segmented the T1 images with the standard unified segmentation routine in 3 classes

(as implemented in SPM5) and with the modified segmentation routine in 4 classes. The segmented classes are almost identical for the two methods, as illustrated in the scatter-plots for GM (or WM) tissue with the standard segmentation and the new segmentation (Figure S2). In addition, we computed the absolute differences between the GM and WM obtained from both segmentations and calculated their mean (±SD) over all voxels for each subject. From this analysis, the maximum mean differences over the 8 subjects in GM and WM probabilities were negligible (maximum = 0.007±0.01 and 0.003±0.008 respectively). This supported a high specificity of this new segmentation (i.e. the extra class was empty when the brain was normal).

3- Influence of the spatial smoothing

We tested the effects of spatial smoothing in all simulated lesions. We compared different full-width-at-half-maximum (FWHM) values of the Gaussian kernel (0mm, 4mm, 8mm, 12mm, 16mm and 20mm). Lesion identification (i.e., outlier identification) was performed on GM and WM images with several FWHM values. For each FWHM value, we assessed Dice's similarity index between each binary lesion map (i.e. thresholded F_{LES}) and the "real" lesion (i.e. the manually defined lesion considered as true positives) using the following formula:

$$Dice = \frac{2 \cdot TP}{2 \cdot TP + FP + FN}$$

where TP, FP, and FN represent the number of true positives, false positives, and false negatives respectively. Because the lesion is a fuzzy set (i.e. F_{LES} contained values between 0 and 1), the Dice index was generated at several *U* thresholds.

We found a direct influence of spatial smoothing on the sensitivity of the method, as illustrated by the Dice similarity index plots (see Figure S3A). At low *U* values (e.g. $U<0.05$), the Dice index was small due to high false positive rates. The maximum value for the Dice index over all *U* values was higher for intermediate/high spatial smoothing (i.e. FWHM > 8mm) than for low smoothing (i.e. FWHM < 8mm). However, over a wide range of intermediate/high *U* values (e.g. $U>0.3$), and except for the unsmoothed images, the Dice index decreased with spatial smoothing (i.e.,

Dice values decreased when FWHM increased from 4mm to 20mm). Intermediate smoothing values (e.g. 8 and 12mm) therefore represented a compromise between the two effects. Moreover, smoothing at 8mm provided a better shape for the fuzzy lesion with accurate definition (sharp boundaries) as shown in Figure S3B (e.g. red curve at 8mm has sharp limits compared to other curves at high spatial smoothing). From this analysis, we suggest that an intermediate smoothing of 8mm FWHM is suitable for the type of lesions tested here.

4- Influence of the U thresholds

As shown in the manuscript, the identified lesion is represented by the fuzzy set F_{LES}. This fuzzy set codes the degree of abnormality of each voxel with continuous values between 0 and 1 (i.e. U values). To generate a binary image of the lesion (i.e., boundaries), the fuzzy set F_{LES} can be thresholded at a given U value. The "binarisation" of F_{LES} obviously depends on the choice of U thresholds. We explored the influence of the threshold on the exact definition of lesion boundaries. Lesion boundaries were assessed and compared topographically at different thresholds for all simulated lesions.

The binarisation effect is illustrated in Figure S4 for two simulated lesions. Low threshold values sometimes led to the identification of regions outside the lesion (e.g., false positives in simulated case 01). Intermediate thresholds of 0.3 or 0.5 provided better results with these simulated lesions. Note that the definition of "exact" lesion boundaries in a mono-spectral mode is difficult even with manual segmentation, because of partial volume effects in T1 images with limited spatial resolution.

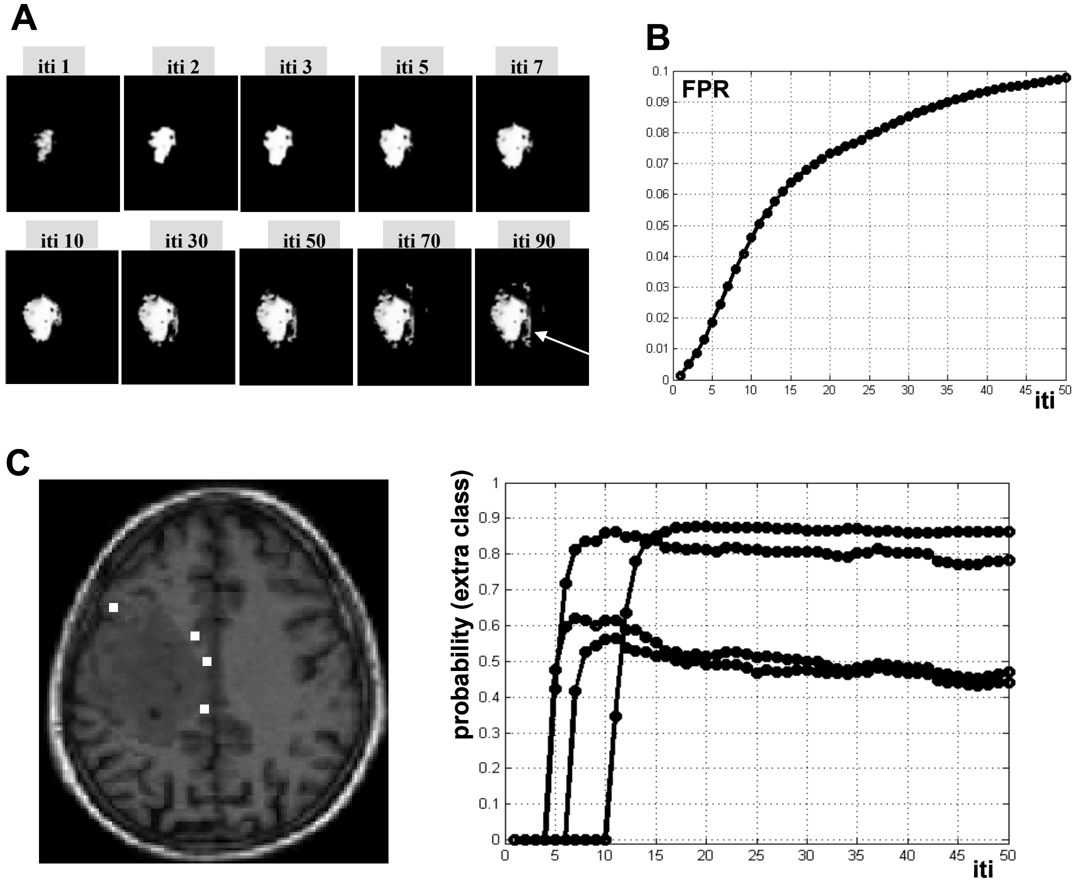

Figure S1. (A) The evolution of empirical priors on the "extra" class, when the number of iterations (i.e., number of segmentation runs) increased from 1 through to 90 (for simulated case 06). The white arrow indicates the misclassification of healthy GM into the extra class for higher iterations. (B) the False Positive Rate (FPR) increased with the number of iterations. (C) Probability values for the extra class in 4 voxels from healthy GM near to the lesion as a function of the number of iterations (right). The location of these voxels is shown in white dots on an axial slice (left). The abbreviation "iti" stands for the number of iterations.

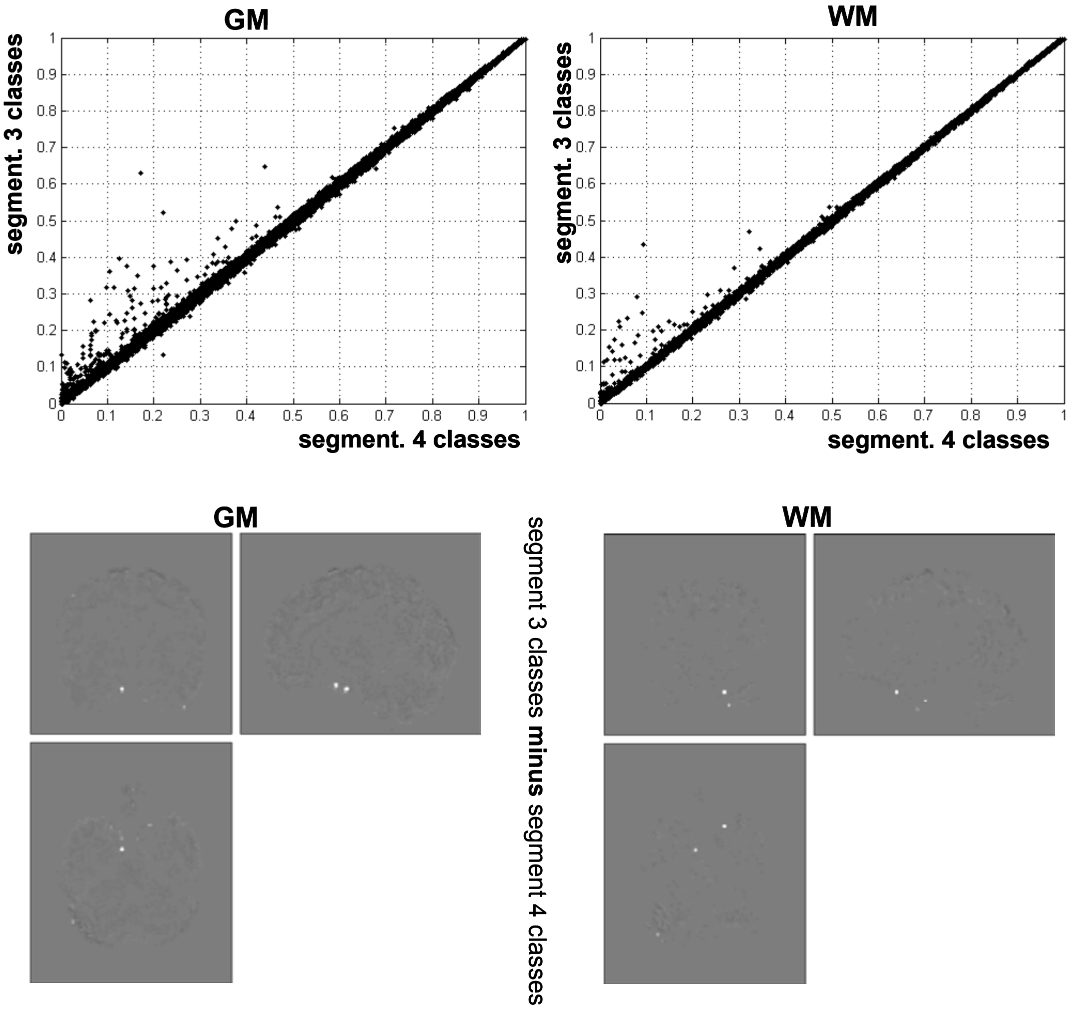

Figure S2. (Top) a scatter-plot of the GM probability (left) and WM probability (right) of each voxel (270,518 voxels) between the standard SPM5 segmentation (in 3 classes) and the modified unified segmentation (in 4 classes). (Bottom) Voxels with high differences (>0.1 in absolute values) between the two segmentations are illustrated for the GM (left) and WM (right). These voxels are located in regions at the edge of the cortex where potential interpolation errors (e.g. during normalisation) are likely to occur. Display = gray scale between -0.1 to +0.1; three orthogonal views (coronal, sagittal, and axial).

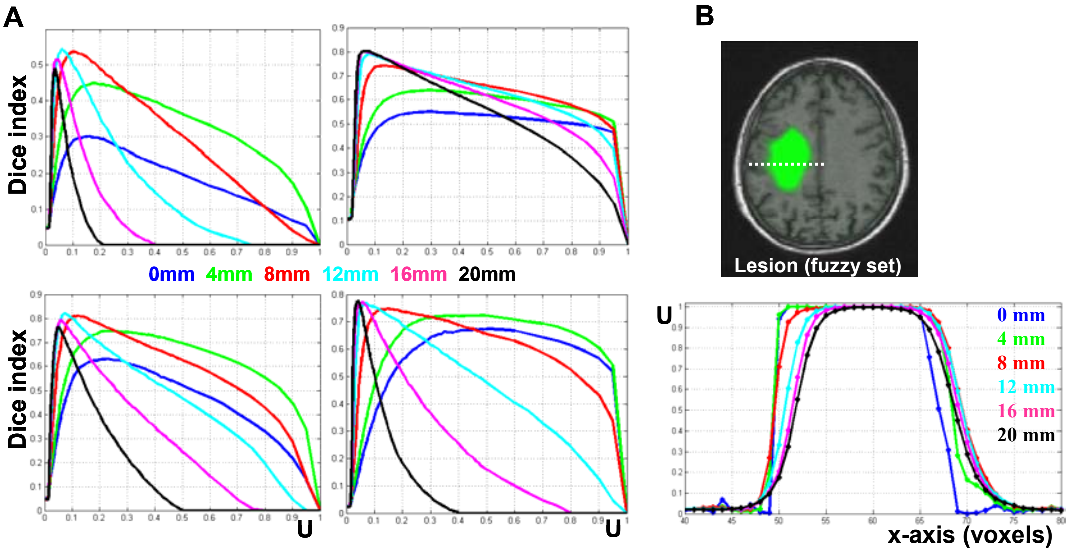

Figure S3. (A) Illustration of the Dice index, computed at different U thresholds (x-axis), for 4 different simulated lesions (simulated cases 02, 06, 09 and 10). All six smoothing levels are shown in different colours. Intermediate smoothing (e.g. 8mm) shows optimum results; where the Dice index is high over a large range of U values. (B) The resulting images illustrate the effects of different levels of spatial smoothing on the definition of lesion boundaries: (top) the fuzzy set of the lesion is shown in green on an axial slice form simulated case 06 and at 8 mm smoothing; (bottom) U values at different smoothing values when moving voxels in the x-direction along the white dashed-line.

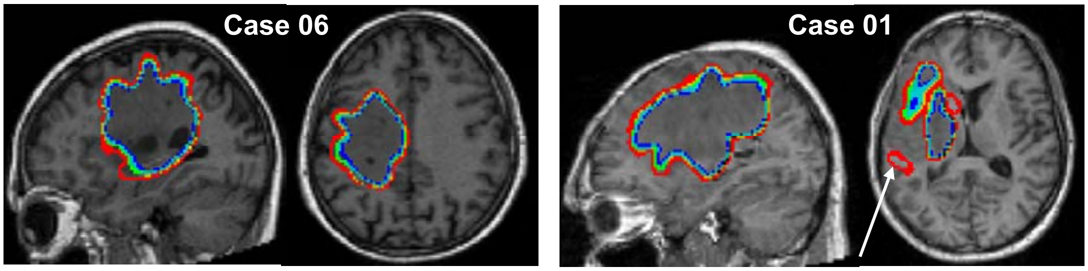

U>0.1; U>0.3; U>0.5

Figure S4. Illustration of lesion boundaries of simulated cases 06 (left) and 01 (right) with a smoothing of 8mm. Lesion boundaries are defined at three different thresholds U>0.1 (red), U>0.3 (green) and U>0.5 (blue). The white arrow indicates the false lesion boundaries of case 01 when using a low threshold (U>0.1).
